# Immunohistochemical localization of mu opioid receptor in the marginal division with comparison to patches in the neostriatum of the rat brain

**DOI:** 10.1186/1423-0127-18-34

**Published:** 2011-06-01

**Authors:** Chuanxing Wang, Si Yun Shu, Zhouyi Guo, Ye Feng Cai, Xinmin Bao, Changchun Zeng, Bingyi Wu, Ziyou Hu, Xuemei Liu

**Affiliations:** 1 College of Biophotonics, South China Normal University, Guangzhou, GD 510631, China; 2 Institute of Cognitive Neuroscience, South China Normal University, Guangzhou, GD 510631, China; 3 First Department of Neurology, Second Affiliated Hospital, Guangzhou University of Traditional Chinese medicine, Guangdong Provincial Hospital of Traditional Chinese Medicine, Guangzhou 510120, China; 4 Research Center of Clinical Medicine, Nan Fang Hospital, Southern Medical University, Guangzhou 510515, China

**Keywords:** Mu opioid receptor, Neostriatum, Marginal division, Patches, Immunohistochemistry, Western blot

## Abstract

**Background:**

Mu opioid receptor (MOR), which plays key roles in analgesia and also has effects on learning and memory, was reported to distribute abundantly in the patches of the neostriatum. The marginal division (MrD) of the neostriatum, which located at the caudomedial border of the neostriatum, was found to stain for enkephalin and substance P immunoreactivities and this region was found to be involved in learning and memory in our previous study. However, whether MOR also exists in the MrD has not yet been determined.

**Methods:**

In this study, we used western blot analysis and immunoperoxidase histochemical methods with glucose oxidase-DAB-nickel staining to investigate the expression of MOR in the MrD by comparison to the patches in the neostriatum.

**Results:**

The results from western blot analyses revealed that the antibody to MOR detected a 53 kDa protein band, which corresponded directly to the molecular weight of MOR. Immunohistochemical results showed that punctate MOR-immunoreacted fibers were observed in the "patch" areas in the rostrodorsal part of the neostriatum but these previous studies showed neither labelled neuronal cell bodies, nor were they shown in the caudal part of the neostriatum. Dorsoventrally oriented dark MOR-immunoreactive nerve fibers with individual labelled fusiform cell bodies were firstly observed in the band at the caudomedial border, the MrD, of the neostriatum. The location of the MOR-immunoreactivity was in the caudomedial border of the neostriatum. The morphology of the labelled fusiform neuronal somatas and the dorsoventrally oriented MOR-immunoreacted fibers in the MrD was distinct from the punctate MOR-immunoreactive diffuse mosaic-patterned patches in the neostriatum.

**Conclusions:**

The results indicated that MOR was expressed in the MrD as well as in patches in the neostriatum of the rat brain, but with different morphological characteristics. The punctate MOR-immunoreactive and diffuse mosaic-patterned patches were located in the rostrodorsal part of the neostriatum. By contrast, in the MrD, the dorsoventrally parallel oriented MOR-immunoreactive fibers with individual labelled fusiform neuronal somatas were densely packed in the caudomedial border of the neostriatum. The morphological difference in MOR immunoreactivity between the MrD and the patches indicated potential functional differences between them. The MOR most likely plays a role in learning and memory associated functions of the MrD.

## Background

The neostriatum in the rat brain has been reported to be divided into two compartments, striosomes/patches and matrix, which contribute to the heterogeneous nature of the neostriatum [1-3]. Pert et al [4] distinguished the "patch" compartment by its dense concentration of opioid receptors in the rat and termed the rest of the surrounding striatal tissue "matrix". The patch-matrix compartment can be recognized on the basis of the expression of several markers, including enkephalin, substance P, calcium-binding protein and opioid receptors. The matrix is enriched in met-enkephalin positive cells [2,5] and acetylcholinesterase expressing cells [3,6]. In contrast, the striosomes/patch compartment is enriched in fibers that are immunoreactive for substance P and leu-enkephalin [7] and calretinin [8].

The marginal division of the neostriatum (MrD) was shown to be located at the caudomedial border of the neostriatum, surrounding the rostrolateral edge of the globus pallidus in the rat brain [9]. The localization of the MrD has been confirmed by other researchers. Schoen and Graybiel found 5'-nucleotidase activity densely expressed in the developing rodent caudoputamen (location of the MrD) association of with dopamine islands and striosomes in rat, but with extrastriosomal matrix in mouse [10]. The staining intensity for the A subtype of α2-adrenergic receptors was higher in the MrD than in the rest of the rat striatum [11]. Most of the neuropeptides and receptors expressed in the MrD were reported to exert influences on learning and memory functions of the brain [12,13]. The MrD has been found to be involved in learning and memory through behavioural studies of rats [14], LTP studies [15] and in functional magnetic resonance image studies of humans [16]. In addition, the MrD was implicated in the modulation of pain by other investigators. Nociceptive neurons were reported to be localized exclusively in the MrD of rat striatum by Chudler and Dong [17] and Chudler et al. [18], using neurophysiological methods, suggesting that the MrD might be involved in pain modulation. The MrD is distinguished from the rest of the neostriatum by its special cytoarchitecture, its neurochemistry, and the efferent connections to the globus pallidus and substantia nigra. Previous immunohistochemical studies on the MrD showed a unique immunohistochemical staining profile by comparison to the rest of the neostriatum. Like the patches, the staining of AChE was weaker in the MrD than in the rest of the neostriatum [9], and a layer of densely packed substance P and leu-enkephalin immunoreactive fibers and terminals was observed in the MrD in rat and cat [19]. However, met-enkephalin immunostaining was reported to be more intensely packed in the rat MrD than in the rest of the neostriatum, which differed from that of the patches but was similar to that of matrix [9].

Mu opioid receptors (MORs) are one member of the seven transmembrane family of G-protein coupled receptors [20-23]. Their activation, by endogenous opioid peptides and exogenous opioid drugs, is intimately involved with a range of physiological processes underlying pain and analgesia, tolerance and dependence, learning and memory, eating and drinking, alcohol, drugs of abuse etc [24]. The distribution of MOR has been extensively studied in the rat striatum through the techniques of binding autoradiography [25-27], in situ hybridization histochemistry [20-23,28-31] and immunohistochemistry [32-37]. These studies have indicated that MOR is preferentially localized to the patches of the rat neostriatum. The "patch" compartment is distinguished from the matrix by its dense concentration of MOR in the rat neostriatum, and the restricted MOR binding pattern is considered one of the striatal markers.

However, the distribution of MOR has not yet been described in the MrD. In this study, we employed western blot analysis and immunoperoxidase histochemical methods with glucose oxidase-DAB-nickel staining to investigate whether MORs also localize to the MrD, and we compared the immunohistochemical distribution of MOR-immunoreactivity in the MrD with that of patches of the neostriatum.

## Methods

### Animals

Experiment were performed on 10 adult male Sprague-Dawley rats (220 g--250 g, Laboratory animal center, Guangzhou University of Chinese Medicine, China) maintained on a 12/12 hours light/dark cycle and were allowed free access to food and water. Experiments were carried out according to a protocol approved by the Animal Care Committee at South China Normal University and in accordance with policies and guidelines of the Chinese Council on Animal Care.

### Antibody

A rabbit polyclonal antiserum raised against a synthetic peptide (aa 384-398) corresponding to the C-terminus of rat MOR1 (Immunostar, Cat. 24216) was used in this study. This commercially available antibody has been extensively used for immunohistochemistry in the rat CNS [32,38-44]. Specificity of the MOR antiserum has been demonstrated previously on the basis of epitope-expressing cell lines, western blotting, and adsorption controls [32,45].

### Western blot

Tissues (the MrD, the hippocampus of rat brains) were minced and homogenized in cold lysis buffer (50 mM Tris pH 7.4, 150 mM NaCl, 1% Triton X-100, 1% sodium deoxycholate, 0.1% SDS, and protease inhibitors cocktail). Protein concentration was determined by the BCA method. Protein samples (40-50 μg) were subjected to SDS-PAGE and transferred onto a PVDF membrane according to the method of Towbin et al [46]. The membrane was blocked with 5% non-fat dry milk in Tris-buffered saline (TBS) for 1 hr, and then incubated with anti-MOR (1:1500 dilution) antibody in TBS containing 0.1% Tween-20 at 4°C overnight. After washing with TBST (20 mM Tris, 140 mM NaCl, 0.1% Tween-20, pH 7.6), immunoreactive bands were detected with goat anti-rabbit IgG conjugated with horseradish peroxidase (1:800) and developed using the ECL detection system. The hippocampus was used as the positive control group.

### Immunohistochemistry

Rats were heavily anesthetized with chloral hydrate (400 mg/kg, i.p.), and perfused through the aortic arch with 200 ml 0.9% saline, followed by 500 ml 4% paraformaldehyde in 0.1M phosphate buffer (PB, pH 7.4) in 20-30 minutes. The brain was removed and post-fixed for 2 hours at 4°C in 4% paraformaldehyde in 0.1M PB, pH 7.4. The brain was then transferred to 30% sucrose in 0.1M PB and stored at 4°C until the tissue had sunk to the bottom of the sucrose solution in the bottle. Tissues were cropped, embedded in Jung Tissue Freezing Medium (Leica, Germany) and gradually frozen at -18°C in a Leica CM 1950 cryostat. Brains were sectioned coronally of 30-μm thickness at -18°C in a Leica CM 1950 cryostat and collected in 0.1M PB, pH 7.4.

Tissues were processed as floating sections. Sections were washed with 0.01M phosphate buffered-saline (PBS, pH 7.4) and incubated in the primary antibody against Mu opioid receptor (1:3500; Rabbit anti-MOR, Immunostar, Cat. 24216) diluted in PBS/0.3% Triton X-100 for 18 hours at 4°C. Unbound primary antibodies were then removed by washing with PBS (pH 7.4) three times. Sections were processed with Rabbit HRP-Polymer Kit (PV-6001, ZSGB-BIO, China) and further treated by the glucose oxidase-DAB-nickel method [47]. The reaction was terminated by three consecutive 0.1M acetate buffer (pH 6.0) washes, after which sections were mounted on gelatin-coated slides. The sections were then treated with graded alcohols, and xylene, placed on coverslips with Neutral balsam, and observed with a Lecia microscope (DM 2500, Germany). In the control experiment, the primary antibody was replaced with 0.3% Triton X-100 in PBS (pH 7.4).

## Results

### Western blot

**Figure 1:**
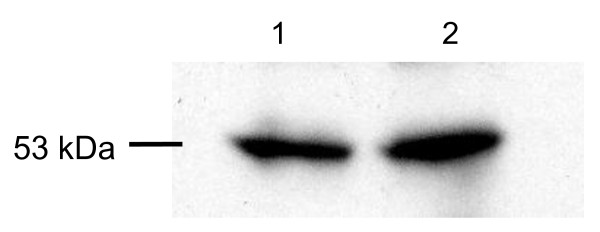
**Immunoblot of SDS extracts of the Marginal division (MrD) and the hippocampus of the rat brain with an anti-peptide antibody**. Lane 1, the MrD; Lane 2, hippocampus (positive control).

Western blot analyses were performed on lysates of the rat MrD and also on the Hippocampal tissue using a polyclonal antiserum against a peptide mapping at the C terminus of MOR. The results revealed an immunoreactive band of about 53 kDa (Figure 1 line1) that corresponds to the de-glycosylated form of MOR [48]. In the positive control group, the positive signal of the specific 53 kDa immunoreactive band was also obtained (Figure 1 line2).

### Overview of the immunohistochemical localization of mu opioid receptor

MOR-immunoreactivity was found unevenly distributing at different levels of the neostriatum. The neuropil labelled with intense MOR immunoreactivity was seen in a dorsoventrally oriented moon-shaped band in the MrD at the caudomedial border of the neostriatum. The mosaic distribution patterns of MOR staining were observed in patches in the rostrodorsal part of the neostriatum. Details of the distribution of MORs in the MrD and the patches of the neostriatum were presented below.

### Immunohistochemical localization of mu opioid receptor in the Neostriatum (excluding the MrD)

**Figure 2:**
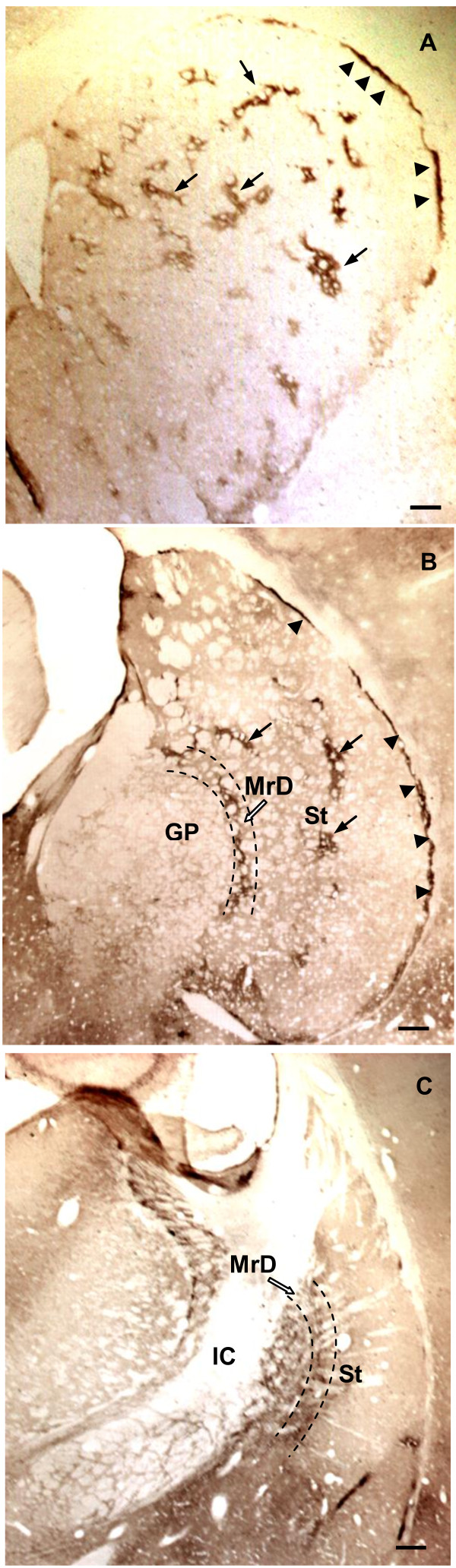
**Comparative distribution of MOR-immunoreactivity at different levels of coronal sections of the rat neostriatum (St): coronal sections stained with anti-MOR and GDN method arranged in a rostrocaudal order**. A: MOR-immunoreactivity was localized in densely stained patches (arrows) and the subcallosal streak (arrowheads) at the rostral part of the St. Patches of MOR-immunoreactivity was most prominent in the rostral, dorsal portion of the neostriatum, which distributed irregularly, exhibiting complex and tortuous fields with multiple extensions. B: At the rostromedial portion of the St, the number of the patches decreased and the staining of MOR-immunoreactivity in the MrD was seen in a moon shape band that parallels with the subcallosal streak. C: At the caudomedial portion of the St, MOR-immunoreactivity was seen densely stained in the band of nerve fibers that arranged in parallels in the MrD. All scale bars: 500 um.

**Figure 3:**
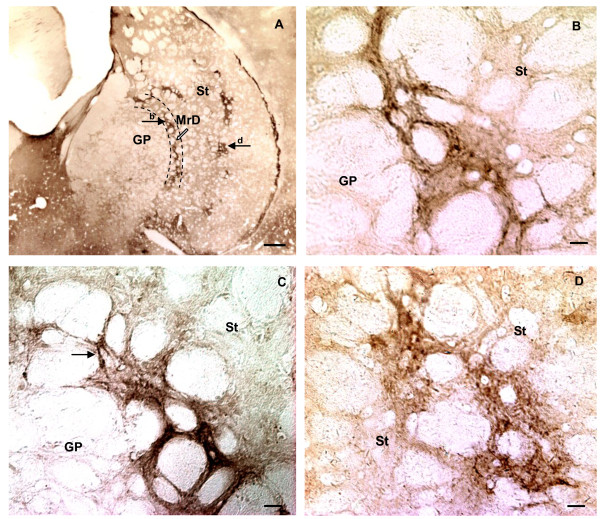
**Localization of MOR-immunoreactivity in medial part of the neostriatum (St) in coronal sections of the rat brain stained with anti-MOR and GDN method**. A: Figure at low magnification illustrating the distribution of MOR-immunoreactivity in rostral part of MrD and the St. MOR-immunoreactivity labelled nerve fibers were densely packed in a moon-like nerve fiber "band" dorsoventrally oriented in the MrD between the St and the GP (arrow indicated by b in A). Patches of MOR-immunoreactivity were localized in the St. B: At higher magnification, fine, punctate MOR-immunoreactivity was seen within the nerve fibers of the "band". C: The fusiform cell body and its processes were also observed among the diffuse, punctate staining in parallel-arranged nerve fibers in the band of the MrD (arrow in C). Several patches of MOR-immunoreactivity were localized in the St, with their extensions dorsoventrally oriented (the arrow indicated by d in A; D). Arrows of b and d refer to areas shown at higher magnification in B and D, Scale bars: A, 500 um; B, C and D, 50 um.

The typical mosaic pattern of distribution of MOR immunoreactivity was observed in the neostriatum (St). Dense MOR immunoreactivity was seen in the patches in the rostrodorsal portion of the neostriatum (arrows in Figure 2A, B) and in the subcallosal streak (arrowheads in Figure 2A, B) that surrounds the outside edge of the neostriatum as well. The immunoreactivity varied at different levels of the neostriatum. This labelling was most prominent at the rostral portion and it was more pronounced rostral-laterally than caudal-medially. At the rostral portion (Figure 2A), abundant patches were distributed irregularly, exhibiting complex and tortuous morphology with multiple extensions. While the patches at the medial portion (Figure 2B) were sparse, small in size and dorsoventrally oriented. No patches were seen in the caudal part of the neostriatum where the MOR immunoreactivity was densely accumulated in the MrD (Figure 2C). At higher magnification fine, diffuse, punctate MOR immunoreactivity was seen within the patches (Figure 3D). The punctate staining of neuronal cell bodies was not observed in the neostriatum.

### Immunohistochemical localization of mu opioid receptor in the marginal division of the neostriatum (MrD)

**Figure 4:**
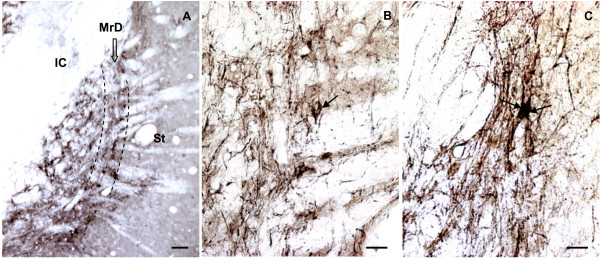
**Photographs of coronal sections of the caudal part of the neostriatum (St) illustrating the presence of MOR in the MrD**. No obvious "patches" of MOR-immunoreactivity was seen. A: A figure at low magnification showed dense accumulation of nerve fibers of MOR-immunoreactivity in the MrD and in the globus pallidus (GP) as well, and weak staining in the rest of the St. B: At higher magnification, the fusiform cell bodies exhibited MOR-immunoreactivity in puncta within parallel-arranged nerve fibers in the MrD (the arrow in B). C: The cell bodies and their dendrites stretched dorsoventrally were showed in the MrD (arrows in C). Scale bars: A, 200 um; B, C, 50 um.

MOR immunoreactivity was concentrated in the dorsoventrally oriented, moon-shaped band that corresponded to the MrD at low magnification of the microscope (Figure 2B, C; Figure 3A; Figure 4A). At higher magnification, dorsoventrally oriented positive MOR immunoreactive dendrites and axons were seen within the MrD (Figure 3B), and a positive MOR-immunostained fusiform cell body was also observed among the labelled nerve fibers (the arrow in Figure 3C).

Dense dorsoventral parallel-oriented MOR-immunostained nerve fibers and terminals were seen concentrated in the MrD at the caudomedial portion of the neostriatum (Figure 2C; Figure 4A). Individual MOR-immunostained fusiform cell bodies were also observed in dorsoventral MOR-immunoreactive nerve fibers that were parallel in their distribution (arrows in Figure 4B, C).

## Discussion

### Morphological characteristics of the MrD in the neostriatum of the rat brain

**Figure 5:**
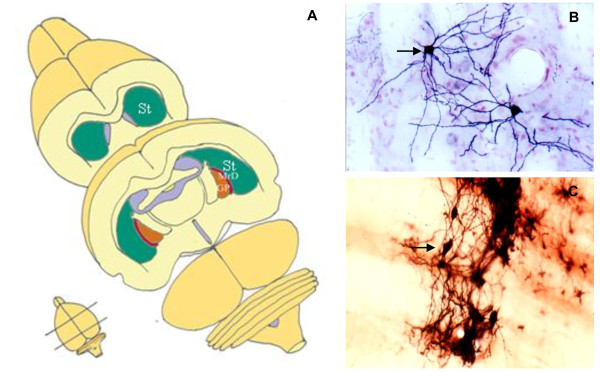
**The location and cellular morphology of the marginal division (MrD)**. A: The MrD is at the caudomedial edge of the neostriatum (St) and surrounding the rostrolateral of the globus pallidus (GP). B: More than 90% neurons in the striatum are medium-sized, round or multipolar neuronal cells with many long dendrites radiating from the neuronal cell bodies. C: The neuronal somata in the MrD are fusiform in shape with their long axes running parallel to the border between the St and the GP.

The MrD is a pan-shaped region within the neostriatum. It localizes at the caudomedial edge of the neostriatum, surrounding the rostrolateral border of the globus pallidus (GP), in the brain of rats (Figure 5A). The MrD can be characterized from the criteria of the rat atlas [49], as well as from its special neuronal morphology (Figure 5B, C), its immunohistochemical characteristics and by the analysis of its specific projection patterns [9].

The rostral part of the MrD appears simultaneously with the appearance of the GP and lies between the caudomedial portion of neostriatum (St) and the rostrolateral border of GP in coronal sections of rat brain. The central part of the MrD is located between the St and GP, leaving the caudal part medial to the caudal-most edge of St where the GP gradually disappears. Morphologically, the neuronal somatas of the marginal division are mostly fusiform in shape, with their long axes running parallel to the border between the striatum and the globus pallidus. Immunohistochemically, the marginal division is lighter in AChE staining without choline acetyltransferase (ChAT)-immunoreacted neurons. It was more intensely stained for substance P and Met-enkephalin-immunoreactive fibers and terminals than the rest of the neostriatum. The efferent fibers of MrD project to the caudal-most part of GP which contains cholinergic neurons of nucleus basalis of Meynert [9,50]. It was demonstrated that the pedunculopontine nucleus gives rise to massive afferent terminals in the MrD, which were seldom found in the rest of the striatum in squirrel monkey [51]. Shammah-Lagnado et al [52] investigated the afferents to the interstitial nucleus of the posterior limb of the anterior commissure in the rat through the use of retrograde (cholera toxin B subunit) tracers. Retrogradely labelled cells are present ipsilaterally in the MrD. This finding indicated that the MrD connected to the interstitial nucleus of the posterior limb of the anterior commissure.

In the present study, the position of the MrD was identified according to its location in the rat atlas, and confirmed by the morphology of individual MOR-immunoreactive fusiform neuronal somatas and the dorsoventral parallel-orientated nerve fibers that were numerous at the caudomedial margin of the neostriatum.

### Existence of MOR in the MrD

In this study, the presence of MOR was firstly described in the MrD by western blot analysis and immunohistochemical methods. Enkephalin, which has a high affinity to MOR and is considered to be one of the endogenous ligands for MOR, was reported to be expressed mostly on the fibers and few on neuronal somatas of the MrD [53]. Electron microscopic analysis of the MrD in the brain of monkeys showed that enkephalin-immunoreactivity was mainly on axons, and these axons formed complex synapses on unlabeled dendrites or axons (Unpublished results). In the present study, MOR immunoreactivity was mostly observed in dorsoventrally oriented nerve fibers and terminals in the MrD. This means that there are most likely interactions between the ENK-immunoreactive nerve fiber terminals and MOR in the MrD. In addition, this distribution of nociceptive neurons was proved in the MrD using a neurophysiological method [18]. As described above, MOR plays key roles in analgesia and also has effects on learning and memory. The presence of both enkephalin and MOR in the MrD suggests that MOR might play a role in the learning and memory functions of the MrD and probably in the pain modulation process. However, definitive evidence for this idea is beyond the scope of this study and will await further experimentation.

### Comparison of MOR-immunoreactivity in the MrD and the neostriatum

Although the distribution of MOR in the striatum has been extensively studied previously, the presence of MOR in the MrD has not been previously examined. In this study, high level MOR immunoreactivity was detected in patches of the neostriatum but with no expression in matrix, as described in some previous published papers [32-37]. We firstly observed strongly labelled MOR-immunoreacted fibers, terminals and individual fusiform neuronal somatas in the dorsoventrally oriented band at caudomedial edge of the neostriatum, which was the location of the MrD. Immunohistochemical features of MOR immunoreactivity in the MrD are different from those of patches in the neostriatum (Table 1). Firstly, the localization of MOR-immunoreactivity in patches and the MrD is different. Positive MOR immunoreactivity are seen in the patches in the rostrodorsal part of the neostriatum, while MOR-immunoreactive fibers, terminals and neurons are observed in the MrD at the caudomedial edge of the neostriatum. Secondly, the morphology of the individual labelled neuronal somata in the MrD is fusiform shaped, whereas the labelled neuronal somata are not seen in patches of the neostriatum. Thirdly, the densely packed MOR-immunoreacted nerve fibers are dorsoventral and oriented in parallel in the MrD, but MOR-immunoreactive nerve fibers are irregularly distributed in mosaic patterns to make the patches in the rostrodorsal part of the neostriatum.

**Table 1:** Comparison of MOR-immunoreactivity between the MrD and patches of the neostriatum

	*patches of the neostriatum*	*the marginal division of the neostriatum*
*Location*	at the rostrodorsal part of the neostriatum	at the caudomedial portion of the neostriatum
*MOR labelled nerve fibers*	irregularly distributed in mosaic distribution patterns	dorsoventral parallel oriented fibers
*MOR labelled neuronal somata*	without seen	fusiform neuronal somatas with and their long axis dorsoventrally oriented

MOR is preferentially localized in patches in the rat neostriatum. Although MOR-immunoreactive areas highly enriched in patches are of interest, but they are not easy to be stereotaxically localized. Manipulation aims to reach patch or matrix compartment are difficult in vivo. The MrD could be stereotaxically identified in vivo from the Atlas of the rat brain. Our data indicate that the MrD is an ideal choice for the study of MOR in the neostriatum in vivo.

## Conclusions

We have for the first time demonstrated the existence of mu opioid receptors (MORs) in the marginal division of the neostriatum by western blot analysis and immunohistochemical methods. The unique morphology of the labelled fusiform neuronal somatas and the dorsoventrally oriented MOR-immunoreactive fibers in the MrD at the caudomedial margin may serve as the markers to distinguish it from patches in the neostriatum. The MOR has been reported to be involved in pain modulation and learning and memory. The pain related neurons have been detected exclusively in the marginal division of the neostriatum. Therefore, MOR is likely to play a role in learning and memory functions of the MrD as well as in pain modulation. The MrD constitutes an ideal region for the study of MOR in the neostriatum, because of its high density of MOR and its consistent reproducible localization in the brain of rat.

## Competing interests

The authors declare that they have no competing interests.

## Authors' contributions

CW participated in the design of the study, carried out the immunohistochemical studies, participated in the western blot analysis and drafted the manuscript. SYS conceived the overall study, directed its design, experiments and coordination, and contributed to important intellectual content and final revision and approval of the manuscript. ZG participated in coordination and helped the experiments. YFC contributed to the scientific discussion. XB directed the immunohistochemical studies. CZ and XL participated in the immunohistochemical experiments and helped to draft the manuscript. All authors read and approved the final manuscript.
